# Long-Term Impact of Recurrent Acute Otitis Media on Balance and Vestibular Function in Children

**DOI:** 10.3390/brainsci14121246

**Published:** 2024-12-12

**Authors:** Mirko Aldè, Pietro Bosi, Stefanie Muck, Thomas Mayr, Paola Di Mauro, Valentina Berto, Beatrice Gaia Aleandri, Francesco Folino, Stefania Barozzi, Diego Zanetti, Paola Marchisio

**Affiliations:** 1 Department of Clinical Sciences and Community Health, University of Milan, 20122 Milan, Italy; mirko.alde@unimi.it (M.A.); beatricegaia.aleandri@studenti.unimi.it (B.G.A.); stefania.barozzi@unimi.it (S.B.); diego.zanetti@unimi.it (D.Z.); 2 Audiology Unit, Department of Specialist Surgical Sciences, Fondazione IRCCS Ca’ Granda Ospedale Maggiore Policlinico, 20122 Milan, Italy; valentina.berto@policlinico.mi.it; 3 Department of Pathophysiology and Transplantation, University of Milan, 20122 Milan, Italy; pietro.bosi@unimi.it (P.B.); francesco.folino@unimi.it (F.F.); paola.marchisio@unimi.it (P.M.); 4 Department of Health, University of Applied Sciences FH Campus Vienna, 1100 Vienna, Austria; stefanie.muck@edu.fh-campuswien.ac.at; 5 Department of Otorhinolaryngology, Head & Neck Surgery, University Clinic St. Poelten, 3100 St. Poelten, Austria; thomas.mayr@stpoelten.lknoe.at; 6 Department of Medical and Surgical Sciences and Advanced Technologies “G.F. Ingrassia”, ENT Section, University of Catania, 95123 Catania, Italy; 7 Pediatric Highly Intensive Care Unit, Fondazione IRCCS Ca’ Granda Ospedale Maggiore Policlinico, 20122 Milan, Italy

**Keywords:** recurrent acute otitis media, vestibular system, balance disorders, children, posturography

## Abstract

Background/Objectives: Recurrent acute otitis media (rAOM) is a common disease in childhood, but its impact on the vestibular system remains poorly understood. The present study aimed to evaluate the long-term effects of rAOM on balance and vestibular function in pediatric patients. Methods: A total of 55 children, aged 8 years (25 males and 30 females), with a documented history of rAOM, no AOM episodes in the past year, and no previous ear surgery were assessed. Static posturography was used to assess postural instability, measuring sway area (SX, mm^2^) under four conditions: eyes open and eyes closed, with and without foam pads. Vestibular function was evaluated using the video head impulse test (v-HIT) to quantify vestibulo–ocular reflex (VOR) gain and corrective saccades across all six semicircular canals. Results: Children with a history of rAOM demonstrated significantly greater postural instability than healthy controls (*p* < 0.001 for all test conditions). The number of AOM episodes was the primary factor influencing balance dysfunction, with children who had more than eight episodes showing the most pronounced deficits in postural stability (*p* < 0.05). In some cases, the v-HIT revealed hypofunction in the right anterior (14.5%), left posterior (7.3%), left lateral (5.5%), left anterior (3.6%), and right posterior (3.6%) semicircular canals. Conclusions: The results of this study suggest that rAOM can lead to lasting balance and vestibular dysfunction, highlighting the importance of early monitoring and potential rehabilitation.

## 1. Introduction

Balance, coordination, and spatial orientation in children are achieved through the integration of visual, proprioceptive/somatosensory, and vestibular inputs. These sensory signals are primarily processed by the vestibular nuclei and cerebellum, which work together to enable coordinated movements and spatial awareness. Disruptions in any of the sensory systems or in their processing can lead to motor and balance difficulties, hindering a child’s ability to perform daily activities such as walking, climbing stairs, or playing with friends [1,2]. Assessing vestibular function in the pediatric population is particularly challenging due to specific factors inherent to children, such as limited attention and cooperation, difficulties communicating symptoms, developmental variations, and emotional barriers [3]. However, in the presence of qualified professionals, the cervical vestibular-evoked myogenic potentials (cVEMPs) can be conducted in children from 3 months of age. In addition, the video head impulse test (v-HIT) of the horizontal semicircular canals has been described as practicable in children as young as 3 months using a remote camera system, although it appears particularly challenging in those under 3 years. The rotary chair test is feasible after 6 months of age, while the v-HIT of the vertical canals and ocular vestibular-evoked myogenic potentials (oVEMPs) can be performed after 2 years of age. In contrast, the functional head impulse test (f-HIT), posturography, and caloric testing can be executed from 5 years onward [4]. In children, balance disorders specifically resulting from vestibular involvement are often associated with genetic syndromes, migraines, sensorineural hearing loss (HL), congenital infections (especially cytomegalovirus), autoimmune diseases, ototoxic drugs, viral infections, and all types of otitis media [5,6,7,8,9,10,11]. Otitis media is a generic term that refers to any condition involving inflammation of the middle ear without specifying its cause or underlying mechanisms [12]. Otitis media with effusion (OME) is defined as inflammation of the middle ear with an intact tympanic membrane (TM), characterized by middle ear effusion (MEE) without signs or symptoms of acute infection [12,13]. Otoscopic examination usually reveals a cloudy and yellowish TM, often accompanied by an air–fluid level. Tympanometry typically shows a flat curve (type B), while hearing evaluations frequently disclose conductive HL that can vary from mild to moderate [14]. Chronic otitis media with effusion (COME) occurs in cases where MEE persists for 3 months or longer after diagnosis [12,14]. Acute otitis media (AOM) is defined as an infection of the middle ear diagnosed by the following criteria: (1) sudden onset: symptoms, such as ear pain, decreased appetite, and fever, emerge abruptly within the past 48 h; (2) signs of inflammation: the TM exhibits marked erythema and a yellowish hue; and (3) the presence of MEE: there is bulging of the TM indicating fluid buildup in the middle ear [12,15]. Alternatively, the presence of otorrhea (ear discharge) due to spontaneous perforation of the TM, not related to otitis externa, is sufficient to confirm AOM on its own [15]. Recurrent acute otitis media (rAOM) is defined as three or more episodes of AOM within a 6-month period or four or more episodes in the past year, with at least one episode occurring in the last 6 months [16]. Chronic suppurative otitis media (CSOM) is a long-standing inflammatory condition affecting the middle ear, characterized by a perforated TM that fails to heal spontaneously. It typically presents with otorrhea, though there may be periods of inactive disease during which the ear is dry. The disease can progressively cause significant HL due to damage to the TM and potential involvement of the ossicular chain [17]. CSOM can also be classified as cholesteatomatous or noncholesteatomatous, depending on the presence or absence of cholesteatoma, respectively [18]. Cholesteatoma is an abnormal accumulation of keratinized squamous debris in the middle ear. It can be present from birth as a result of residual embryonic ectodermal tissue (*Congenital cholesteatoma*), or it may develop later in life. Acquired cholesteatomas can arise from an epitympanic retraction pocket (Primary-acquired cholesteatoma), often due to chronic Eustachian tube dysfunction, or from a perforated TM (Secondary-acquired cholesteatoma) [19]. In some cases, cholesteatoma may also occur as a complication of ear surgery (*Iatrogenic cholesteatoma*) [20].

The impact of MEE on balance is well-documented, as it increases pressure in the middle ear, affecting the vestibular organs and causing symptoms such as vertigo, dizziness, and unsteadiness [10]. However, the long-term effects of otitis media after its resolution are not fully understood. To address this gap, we decided to conduct a study aimed at evaluating the lasting effects of rAOM on balance and vestibular function in children with no episodes of otitis media in the past year.

## 2. Materials and Methods

### 2.1. Setting

The present study was conducted at the Pediatric Outpatient Audiology Clinic of the Fondazione IRCCS Ca’ Granda, Ospedale Maggiore Policlinico of Milan, Italy, from 1 May 2023 to 30 June 2024. This tertiary-level audiology center is dedicated to the diagnosis, treatment, and rehabilitation of audiological and vestibular disorders in children.

### 2.2. Participants

Children were included in this study if they met the following criteria:

- Age of 8 years;
- Documented history of rAOM, defined as at least four episodes of AOM within a single year;
- No episodes of otitis media (OME, AOM, or CSOM) in the 12 months prior to this study.

All diagnoses of AOM and their treatments had been made by experienced pediatricians at the Fondazione IRCCS Ca’ Granda, Ospedale Maggiore Policlinico of Milan.

The following conditions were grounds for exclusion from this study:

- Any congenital malformations or diseases affecting the outer, middle, or inner ear;
- History of ear surgery, including myringoplasty, tympanoplasty, exploratory tympanotomy, stapedectomy, myringotomy, and tympanostomy tube placement;
- Congenital cytomegalovirus infection;
- Genetic syndromes or mutations known to be associated with HL;
- Clinical evidence of otitis media (OME, AOM, or CSOM) at the time of this study;
- Recent head trauma, exposure to loud noises, or use of ototoxic drugs;
- Any systemic diseases;
- Any neurological disorders;
- Any musculoskeletal disorders;
- Any ocular or visual disorders.

### 2.3. Clinical Assessments

Each participant underwent a comprehensive audio-vestibular evaluation, which included the following components:

- Detailed medical history: a thorough medical history was taken, focusing on the total number of AOM episodes, age at first diagnosis of AOM, and previous presence of otorrhea.
- Otomicroscopy: this allowed for detailed visualization of the external auditory canal and TM, helping to exclude any external or middle ear pathologies.
- Pure-tone audiometry (125 Hz to 8000 Hz): air and bone conduction audiometry was conducted using the Piano clinical audiometer by Inventis (Padua, Italy) to assess the presence of conductive, sensorineural, or mixed HL.
- Tympanometry: this was performed using the Clarinet clinical tympanometer by Inventis (Padua, Italy) to assess the mobility of the TM.
- Spontaneous nystagmus assessment: This was undertaken using GIMA Frenzel glasses with fixed, biconvex lenses of 18.0 diopters (Gessate, Italy). These lenses eliminate visual fixation, enabling clear observation of eye movements.
- Static posturography: This was conducted using the SVeP (Standard Vestibology Platform) by Politecnica (Modena, Italy), a professional stabilometric system used for postural analysis. The platform is a stable force plate (50 cm × 50 cm × 7 cm, 12 kg) with three strain-gauge force transducers arranged in an equilateral triangle (400 mm). Tests were performed with a fixed visual target at a distance of 1.20 m at eye level in a quiet room. Children stood barefoot on the platform with their arms by their sides, following strict foot placement instructions.

The sway area, SX (mm^2^), was analyzed across four test conditions, each lasting 52 s:

- Eyes open (EO): firm surface with eyes open, utilizing all sensory inputs;
- Eyes closed (EC): firm surface with eyes closed, removing visual input;
- PAD EO: foam pads with eyes open, reducing somatosensory input;
- PAD EC: foam pads with eyes closed, removing visual input and reducing somatosensory input.

The sway area (SX) in static posturography refers to the area covered by the body’s center of pressure (COP) as it moves to maintain balance. It is typically measured in square millimeters (mm^2^) and represents the surface area of the confidence ellipse that contains 90% of the COP positions sampled during the test [21].

Video head impulse test (v-HIT): This test assessed the vestibular system’s function, measuring the gain of the vestibulo–ocular reflex (VOR) across all six semicircular canals (horizontal, anterior, and posterior). The ICS^®^ Impulse device from Natus Medical Incorporated (Middleton, WI, USA), equipped with Otosuite software 4.10 Build 1341, was used for testing [22]. Each child wore tightly fitting goggles equipped with a high-speed camera. During the test, the examiner rapidly moved the child’s head in small, quick turns along the planes of the horizontal, anterior, and posterior semicircular canals while the child focused on a flashing light target [4].

Specifically, the mean (±SD) sway area values of our 8-year-old children with a history of rAOM were compared with the corresponding mean values of healthy peers (in accordance with already validated posturographic parameters) [21]. In addition, among our patients with a history of rAOM, possible differences in mean (±SD) sway area values were evaluated according to sex, number of AOM episodes, age at diagnosis of the first AOM episode, and the presence of otorrhea. Finally, the prevalence of hypofunction in the six semicircular canals, defined by the presence of both reduced VOR gain (<0.7) and corrective saccades (covert or overt) in the v-HIT, was calculated, with both criteria being required for the diagnosis [23,24].

This study followed the World Medical Association’s Declaration of Helsinki and received approval from the local ethics committee.

### 2.4. Statistical Analysis

Statistical analyses were performed using Stata 17 (StataCorp., College Station, TX, USA, 2021). The t-test for independent samples was used to compare mean sway area values (SX, mm^2^) between children with a history of rAOM and healthy controls. Multivariate analysis of variance (MANOVA) was also conducted to confirm the results. A *p*-value of less than 0.05 was deemed to indicate statistical significance.

## 3. Results

Overall, 55 pediatric patients aged 8 years (mean age: 101.1 ± 3.2 months) met the inclusion criteria. All patients enrolled in this study had normal hearing thresholds (based on the pure-tone average at 500, 1000, 2000, and 4000 Hz) and type A tympanograms. Three of them exhibited first-degree horizontal right-beating nystagmus, detected by Frenzel goggles during the spontaneous nystagmus evaluation. Neurological examinations were negative, ruling out any central nervous system lesions, such as brainstem or cerebellar dysfunctions. All children reported only occasional mild self-perceived dizziness and played sports regularly, participating in activities at least once a week. The characteristics of the study population are summarized in Table 1.

The control group included 38 healthy children (21 boys and 17 girls), all 8 years old, who had previously undergone the same static posturography test with validated parameters that demonstrated excellent test–retest reliability [21].

Table 2 shows that children with a history of rAOM had significantly higher mean sway area values compared to healthy children (without a history of rAOM) for all four static posturography conditions (EO, EC, Pad EO, and Pad EC) (Table 2).

Table 3 reveals that children with more than eight episodes of AOM had significantly higher mean sway area values. In contrast, sex, age at the first episode of AOM, and the presence of otorrhea did not affect sway area values. This was observed for all four static stabilometry conditions (EO, EC, Pad EO, and Pad EC) (Table 3).

Overall, multivariate analysis confirmed that, when considering the combined effect of all variables, only the number of AOM episodes (>8) had a significant effect on increasing the mean sway area values (*p* = 0.03).

The prevalence of hypofunction in the six different semicircular canals, defined by reduced VOR gain and the presence of corrective saccades, is shown in Table 4. All three patients with first-degree horizontal right-beating nystagmus showed left lateral semicircular hypofunction when assessed using the v-HIT.

## 4. Discussion

The present study evaluated, using static posturography and the v-HIT, the long-term outcomes of rAOM on balance and vestibular function in pediatric patients. Interestingly, children with a history of rAOM had significantly higher mean sway area values compared to healthy controls, indicating much greater postural instability and unsteadiness during all test conditions. 

The potential links between otitis media and balance disorders have been proposed since 1977 [11]. A longitudinal cohort study by Aarhus et al. found that CSOM and HL resulting from rAOM during childhood predispose to an increased risk of dizziness in adulthood [25]. Specifically, Monsanto et al. showed that patients with CSOM had a higher prevalence of dysfunctions in clinical vestibular function tests and poorer postural control compared to healthy individuals [26]. These findings were also confirmed by a more recent study by Abdelmotaleb et al. [27]. The clinical symptoms of imbalance may be attributed to the reduction in the number of vestibular sensory cells and dark cells observed in some patients with CSOM, especially in more advanced stages [28,29]. As a result, abnormalities in the function of the saccule, utricle, and semicircular canals following CSOM can be detected during vestibular assessments performed with cVEMPs, oVEMPs, and the v-HIT, respectively [27,30]. In particular, Tomaz et al. demonstrated that patients with CSOM present a higher prevalence of corrective saccades compared to the non-suppurative and control groups [31]. It should also be kept in mind that CSOM can potentially lead to serious complications such as suppurative labyrinthitis, labyrinthine fistula, or cerebellar abscess, all of which require prompt diagnosis and treatment to minimize consequences [32,33,34]. Tailor et al. suggested that among patients with CSOM, those with more severe HL are at a higher risk of experiencing dizziness or balance disorders [35]. Similarly, Rehagen et al. demonstrated that children with conductive HL caused by OME exhibit more oculomotor abnormalities, indicative of vestibular dysfunctions, than their peers with normal hearing [36]. However, in our study, the mean sway area values were significantly elevated despite normal hearing thresholds, indicating that balance disorders may persist regardless of the final hearing status. It is important to underline that the presence of vestibular disorders, whatever the degree of conductive HL, should be routinely investigated and can help ENT (Ear, Nose, and Throat) specialists determine the need for early surgical intervention, such as myringotomy or placement of tympanostomy tubes, in children with recurrent or chronic MEE. This means that abnormal vestibular test results can strengthen the indication for middle ear surgery to reduce immediate effects and prevent potential long-term balance problems [37,38]. Bista et al. observed a substantial improvement in vestibular function 3 months following myringotomy and tympanostomy tube placement, as reflected by a significant decrease in mean sway velocity on computerized static posturography [39]. Cohen et al. also found that patients undergoing bilateral myringotomy with tube placement showed a gradual decrease in sway velocity values on computerized dynamic posturography [40]. This change was statistically significant compared to control subjects at the 6-month evaluation by Cohen et al., suggesting that the surgical procedure may play a positive role in improving balance and postural stability over months in children with COME [38,40]. Indeed, a very recent study by Kaplan, conducted using computerized dynamic posturography, confirmed both that children with OME present vestibular impairment compared to the general population and that remarkable improvement in vestibular function can be achieved following tympanostomy tube placement [41]. However, although middle ear drainage can improve stabilogram parameters, recovery is often incomplete [42], and long-term negative effects on vestibular function and balance control may persist [43,44]. In particular, Pazdro-Zastawny et al. found that pediatric patients with a history of COME who had undergone bilateral tympanostomy tube placement 5 years earlier showed significantly higher static posturographic parameters in both EO and EC conditions. In addition, these patients demonstrated a higher prevalence of pathological findings on electronystagmography [43]. Sabir et al. also observed that children aged 10 to 12 who had undergone tympanostomy tube placement and/or had three or more ear infections before age 5 exhibited significant impairments in postural stability [44]. The results of our study, which specifically evaluated 8-year-old children with a history of rAOM who had not undergone ear surgery, confirm a lasting impact of ear infections on balance and vestibular function in pediatric patients, especially those with more than eight previous episodes of AOM. Interestingly, consistent with reports from other authors, no child complained of vertigo or daily debilitating dizziness, making the diagnosis of vestibular and balance disorders subtle and challenging in these patients [11,43]. The paucity of reported vestibular symptoms among children who have experienced multiple episodes of otitis media, despite evidence of balance disorders in clinical assessments, may be attributed to various factors. For instance, pediatric patients often have greater difficulty recognizing or articulating feelings of imbalance, while parents tend to ignore or minimize these symptoms [11,41]. However, regardless of being accompanied by MEE, Eustachian tube dysfunction is considered a non-negligible cause of balance disturbances in children, and several pathophysiological mechanisms have been proposed [45]. The Eustachian tube connects the middle ear to the nasopharynx, allowing for pressure equalization. If it fails to open properly, such as during upper respiratory tract infections, negative fluid pressure can develop in the middle ear. This pressure imbalance can not only interfere with normal hearing function but also affect the vestibular system, which is particularly sensitive to fluctuations in pressure [46]. In particular, studies involving animal models demonstrated that variations in middle ear pressure can result in the shifting of the round and oval windows. This displacement triggers secondary movements of the labyrinthine fluids, which can disrupt the normal fluid dynamics in the inner ear and potentially impact balance and vestibular function [47,48]. As a matter of fact, unequal pressures in the middle ear are the underlying cause of *alternobaric vertigo*. This condition occurs when there is a pressure differential between the left and right middle ears, causing discrepancies in vestibular system perception that can lead to vertiginous symptoms. Such imbalances often stem from inadequate equalization of the Eustachian tube, especially during transitions from higher- to lower-pressure environments. Various factors can heighten the risk of this condition, including recent upper respiratory infections and abnormalities in Eustachian tube anatomy [49]. Another plausible theory put forward by Golz et al. argues that toxins from the middle ear can enter the inner ear fluids through the round window and lymphatic and venous systems, resulting in serous labyrinthitis [50]. In that regard, Kaya et al. identified a link between serous labyrinthitis and silent otitis media through an accurate examination of human temporal bones. This association was found to cause irreversible changes, including damage to outer hair cells and the development of cochlear hydrops [51]. These findings may elucidate the long-term balance and vestibular disorders diagnosed in some patients with a history of recurrent or persistent MEE [11,43,44,52]. Another possible explanation involves changes in the ionic channels of kinocilia and stereocilia due to abnormal ion transfer induced by otitis media, thereby altering the response of vestibular receptors [41,44,45]. Therefore, these hypotheses could also account for the hypofunction of the semicircular canals observed in some individuals within our cohort through the v-HIT despite the absence of subjective symptoms. In fact, prolonged ear pressure imbalances, toxins, or changes in ionic channels could lead to lasting negative effects on the vestibular system, especially when these disruptions in the middle ear persist over an extended period, as in patients with more frequent AOM episodes.

It is essential to note that interpreting nystagmus in children requires caution, as small-amplitude first-degree nystagmus may be physiological, particularly when observed at extreme gaze positions. However, all three of our patients with first-degree horizontal right-beating nystagmus showed reduced VOR gain and corrective saccades in the left lateral semicircular canal during the v-HIT, suggesting left-sided vestibular hypofunction. In any case, irrespective of the presence of spontaneous nystagmus, a relevant percentage of semicircular canal hypofunction was detected in our investigation, supporting the hypothesis of persistent vestibular impairment in children with a history of rAOM.

Specifically, our study provides further evidence of the impact of otitis media on balance and vestibular function, highlighting that rAOM can cause long-lasting negative effects that persist well beyond the resolution of acute episodes. Utilizing precise assessments, such as static posturography and the v-HIT of the horizontal and vertical semicircular canals, our findings underscore the critical need for targeted rehabilitation exercises, even when overt clinical symptoms are absent. A tailored approach is particularly important for young children, as they need to reach all their motor development milestones. Rehabilitation should include adaptation exercises to enhance gaze stability, habituation exercises to alleviate dizziness by gradually exposing them to movements that trigger symptoms, and substitution strategies to encourage reliance on visual and proprioceptive cues [4].

### Limitations and Future Prospects

Although our findings are noteworthy, this study has some limitations. First, the analysis included a relatively small cohort of pediatric patients from a single hospital, which may restrict the applicability of the results. Moreover, the use of the v-HIT as one of the primary diagnostic tools may have affected the accuracy and interpretation of the results, as it is less sensitive than caloric tests in detecting minor vestibular function impairments and may generate artifacts, especially in uncooperative children.

The non-inclusion of cVEMPs and oVEMPs in the vestibular assessment protocol is also a notable drawback, as these tests could have provided important insights into the functionality of the saccule and utricle. Indeed, since the v-HIT primarily assesses the function of the semicircular canals and does not provide information on the otolith organs, it may not have offered a complete picture of vestibular function.

The decision to include only children aged 8 may also be considered limiting, but it was made to minimize age-related differences in postural parameters, thereby enhancing the reliability of the results. Future developments of this study could prioritize expanding the age range of participants, conducting a multicenter trial, and increasing follow-up duration to strengthen the robustness of the outcomes.

## 5. Conclusions

As far as we know, this study is the first to specifically evaluate 8-year-old children with a documented history of rAOM and no previous ear surgery, using both static posturography and v-HIT assessments. Our findings demonstrate that pediatric patients with rAOM experience persistent balance impairments, even in the absence of active infection. Indeed, these children exhibited significantly greater postural instability than healthy controls, with the number of AOM episodes being the primary factor influencing balance dysfunction. Additionally, some children showed reduced VOR gain when assessed using the v-HIT, suggesting vestibular involvement. These results highlight the potential long-term impact of rAOM on balance and vestibular function, underscoring the need for early monitoring and rehabilitation. Further research is needed to better elucidate the underlying relationship between otitis media and balance disorders.

## Figures and Tables

**Table 1:** Characteristics of the study population.

Variables	N (%)
Sex	
Male	25 (45.5)
Female	30 (54.5)
Number of episodes of acute otitis media	
4–8	24 (43.6)
>8	31 (56.4)
Age at first diagnosis of acute otitis media	
<1 year old	23 (41.8)
>1 year old	32 (58.2)
Otorrhea	
No	26 (47.3)
Yes	29 (52.7)
TOTAL	55 (100.0)

**Table 2:** Comparison of mean values (±standard deviation) of sway area (SX, mm^2^) between children with a history of recurrent acute otitis media and healthy children.

	Healthy (n = 38) [21] (SX, mm^2^)	History of Recurrent Otitis Media (n = 55) (SX, mm^2^)	*p*-Value
Test			
Eyes open (EO)	364.3 ± 201.2	557.9 ± 153.2	<0.001 *
Eyes closed (EC)	564.4 ± 337.7	792.1 ± 275.0	<0.001 *
Pad EO	445.4 ± 314.6	751.2 ± 235.7	<0.001 *
Pad EC	602.9 ± 442.0	1032.4 ± 309.7	<0.001 *

* Statistically significant.

**Table 3:** Comparison of mean values (±standard deviation) of sway area (SX, mm^2^) between children with a history of recurrent acute otitis media according to sex, number of acute otitis media episodes, age at first acute otitis media episode, and presence of otorrhea.

	Sex			Number of Episodes of Acute Otitis Media			Age at First Diagnosis of Acute Otitis Media			Otorrhea		
Test	Male	Female	*p*-Value	4–8	>8	*p*-Value	<1 Year	>1 Year	*p*-Value	No	Yes	*p*-Value
Eyes open (EO)	591.3 ± 144.0	530.0 ± 157.4	0.14	505.0 ± 125.1	598.8 ± 162.1	0.02 *	589.0 ± 159.9	535.5 ± 144.4	0.2	544.6 ± 170.8	569.8± 137.4	0.55
Eyes closed (EC)	849.7 ± 315.2	744.1 ± 231.0	0.16	703.6 ± 170.6	860.6 ± 320.4	0.03 *	811.4 ± 239.1	778.2 ± 301.1	0.66	791.3 ± 333.0	792.8 ± 216.3	0.98
Pad EO	805.6 ± 234.3	705.8 ± 231.0	0.12	651.0 ± 153.0	828.7 ± 260.5	0.005 *	803.4 ± 236.7	713.7 ± 231.4	0.16	733.8 ± 262.1	766.8 ± 212.8	0.61
Pad EC	1107.9 ± 338.5	969.5 ± 273.5	0.1	899.9 ± 202.2	1134.9 ± 341.2	0.004 *	1079.5 ± 252.7	998.5 ± 344.9	0.35	995.0 ± 357.0	1065.9 ± 262.2	0.40

* Statistically significant.

**Table 4:** Prevalence of hypofunction of the six different semicircular canals assessed using the video head impulse test (v-HIT).

Semicircular Canals	Vestibular Hypofunction, N (%)
Right Lateral (RL)	0 (0.0)
Left Lateral (LL)	3 (5.5)
Right Anterior (RA)	8 (14.5)
Left Posterior (LP)	4 (7.3)
Left Anterior (LA)	2 (3.6)
Right Posterior (RP)	2 (3.6)

## Data Availability

The data presented in this study are available on request from the corresponding author due to privacy concerns.
